# Hepatitis A and B immunity and vaccination willingness among special school employees in Rhineland-Palatinate

**DOI:** 10.3389/fpubh.2025.1657353

**Published:** 2025-11-10

**Authors:** Peter Kegel, Felix Gössler, Nico Schmitz, Pavel Dietz, Stephan Letzel, Elisabeth Diehl

**Affiliations:** 1 Institute of Occupational Social and Environmental Medicine, University Medical Center of the Johannes Gutenberg University Mainz, Mainz, Germany; 2 Institute for Teachers’ Health at the Institute of Occupational, Social and Environmental Medicine, University Medical Center of the Johannes Gutenberg University of Mainz, Mainz, Germany

**Keywords:** special schools, occupational medicine, infection risk, hepatitis A and B, vaccination, teachers

## Abstract

**Introduction:**

Employees at special schools face elevated risks of hepatitis A and B (HAV/HBV) due to close contact with pupils requiring personal care. Evidence on immunity and vaccination uptake in this occupational group is limited.

**Methods:**

Data from 1,742 employees at special schools in Rhineland-Palatinate, Germany (2021–2023), were collected through online anamnesis forms, selected to ensure efficiency and effectiveness, as well as medical evaluations, vaccination records, and anti-HBs testing during mandatory occupational health care. Self-assessed infection risk, HAV/HBV immunity, and vaccination acceptance rates were analyzed using descriptive statistics and logistic regression.

**Results:**

74% of the participants (83.5% female; median age: 43.7 years; 33.3% teachers, 62.2% educational specialists, 2.2% trainees, 2.4% others) completed the online anamnesis; 79% reported an increased occupational infection risk. Medical assessments confirmed HAV immunity in 54% and HBV immunity in 59%. Despite this awareness, vaccination gaps persisted: 58% of all employees received a recommendation for HAV and/or HBV vaccination, but only about half accepted it during the occupational health consultation. Younger age was the only significant predictor of vaccine acceptance (aOR 0.968 CI [0.952, 0.985]; *p* < 0.001).

**Discussion:**

Employees at special schools perceive a high risk of infection, yet substantial gaps in HAV and HBV immunity remain. Despite counseling, vaccination uptake was modest, with younger staff more likely to accept. The discrepancy between high perceived risk and low uptake suggests barriers such as vaccine hesitancy, distrust, or convenience factors. Moreover, the mismatch between self-reported and confirmed immunity underscores the importance of systematic medical examinations. Occupational health care offers a key opportunity for targeted pre-employment vaccination and education, particularly for older employees.

## Introduction

Teachers and educational specialists at special schools for disabled pupils are exposed to distinct stresses and biological hazards due to close contact with pupils in special needs care. This particularly applies to schools focused on holistic and motor development (in Germany, Förderschulen mit dem Förderschwerpunkt Ganzheitliche und Motorische Entwicklung, Förderschulen G und M). Activities, such as personal hygiene, eating, toileting, catheterization and probing expose staff to body fluids for instance as blood or bodily excretions.

Surveys conducted by the Institute for Teachers` Health (Institut für Lehrergesundheit) in 2012 (1) and 2016 (2) found an increased infection risk in these schools, particularly for viral hepatitis A and B (HAV and HBV). Although exact risk occupational infection rates with inherent risks for teachers and educational specialists in special schools are unavailable, the literature suggests elevated HBV seroconversion rates among caregivers of disabled people and teachers. However, distinguishing between work-related and non-work-related sources of infection remains challenging (3, 4). Studies indicate a higher prevalence of anti-HBc-positive individuals among people with disabilities compared to the general population (3, 5).

Although HAV infections typically resolve, severe and fulminant courses occur, particularly in individuals with pre-existing conditions (6). Despite the low prevalence of HBV in the German population, it remains crucial to protect those with higher exposure risks, as HBV can become chronic and lead to hepatocellular carcinoma. Vulnerable groups, such as immunosuppressed individuals, pregnant women, and those with underlying health conditions require targeted immune protection (7). The Standing Committee on Vaccination (Ständige Impfkommission, STIKO) at the Robert Koch Institute recommends the hepatitis B vaccination at the employer’s expense in the case of occupational risk of infection.

Occupational health management in these workplaces must prioritize employee protection through education on infection transmission, protective measures, and vaccination offers. Optimizing preventive efforts requires understanding employees’ immune status and their willingness to be vaccinated. However, limited data exist on immunity levels, and no data is available on vaccination willingness among special school employees.

Occupational physicians play an important role in prevention by offering counseling and vaccinations as part of occupational health care. In Germany, occupational health screenings are legally mandated under the ordinance on preventive occupational health care (Verordnung zur Arbeitsmedizinischen Vorsorge, ArbmedVV) (8). Preventive care is categorized as mandatory, optional, or recommended.

Based on earlier risk assessments (1, 2), occupational health care for biological agents became mandatory in 2021 for special schools with a focus on holistic and motor development in Rhineland-Palatinate. The Institute for Teachers’ Health, commissioned by the Ministry of Education, coordinates this care, which includes medical history, counseling, and vaccination offers. Beyond legal obligations, empirical data are needed to understand actual immunity gaps and vaccination behavior.

The aim of this study was to assess self-perceived infection risk, HAV/HBV immunity, and vaccination acceptance among special school employees in Rhineland-Palatinate.

## Materials and methods

This study is based on occupational health care data collected from 40 special schools for disabled pupils with a focus on holistic and motor development in Rhineland-Palatinate over a 2-year period (April 2021–November 2023).

### Research questions

The following research questions will be addressed:

*RQ1*: How do employees perceive their exposure to biological agents at special schools, and are there differences between occupational groups?

*RQ2*: What is the immune status of employees regarding HAV and HBV, and how does self-assessment compare with medical evaluations based on certificates or serology?

*RQ3*: Are there differences in HAV or HBV immune status between different occupational groups (e.g., teachers vs. educational specialists)?

*RQ4*: Which personal and professional factors influence the decision to follow a doctor’s recommendation for HAV or HBV vaccination?

### Online anamnesis form

To maximize the efficiency and to reduce consultation time of on-site occupational health care, all participants were asked to complete an online anamnesis form prior to undergoing occupational health care. The online form was conducted using LimeSurvey software in compliance with data protection and medical confidentiality requirements. The questionnaire covered occupational and medical history and included screening questions regarding work-related exposure to biological agents. A secure link to the online form was sent to participants through their school administration. Table 1 provides an overview of the questions used to assess individual work-related risks associated with biological agents.

**Table 1:** Questions on work-related risks related to contact with biological agents.

Question	Answer option
Are you at increased risk of infection in your professional life compared to your everyday life? (e.g., nursing care, contact with bodily fluids, first responder, etc.)	Yes = 1 No = 2 No answer = 3
Do you have proven immunity to HAV/HBV? (vaccination, previous infection, or blood test)	Yes = 1 No = 2 No answer = 3
How often do you experience the following in the course of your work? Close physical contact with students (e.g., providing assistance, comforting, blowing one’s nose) Treating injuries (e.g., first-aid) Helping pupils to use the toilet or take care of their bodies Changing or diapering pupil (after wetting or soiling) Assisting pupil with eating Assisting pupil with taking medication Being scratched, bitten, or spat on by pupil Catheterization/probing of pupil	Never = 1 Rarely = 2 Occasionally = 3 Often = 4 Unclear = 5 No answer = 6
Do you have suitable protective equipment (e.g., protective gloves) and hand sanitizer available for care activities?	Yes = 1 No = 2 No answer = 3
Have you had a needlestick injury or similar injury that could transmit pathogens at work?	Yes = 1 No = 2 No answer = 3

### Occupational health care content

The occupational health care process focused on the interaction between an individual’s occupation and health. Occupational, medical, and family histories were recorded, vaccination books were checked and, if necessary and desired, physical examinations, vaccinations, or blood tests for HBV serostatus (HBs antibodies) were conducted. If the online anamnesis form has been previously filled out and submitted, they were reviewed during consultations. If not, relevant information was collected during the consultation.

### Medical evaluation of HAV and HBV immune status

Medical evaluation of HAV and HBV immune status followed the guidelines of the Standing Committee on Vaccination (9):

Immunity to HAV was considered present with at least two documented HAV vaccinations spaced at least 6 months apart, or the equivalent for combination vaccines. Routine antibody testing was not conducted. Prior HAV infection without documentation was not considered as sufficient protection.

Immunity to HBV was considered present with evidence of baseline immunization with HBV vaccines or a combination vaccine and a subsequent positive antibody test (anti-HBs > 100 IU/L). If the antibody levels were between 10 and 100 IU/L, up to six booster vaccinations were administered, with antibody-titer checks after each dose. Unverified reports of vaccinations were not considered sufficient proof of immunity. (Note: The assessment of HBV immunity followed the recommendations of the Standing Committee on Vaccination, which require an anti-HBs antibody level of ≥100 IU/L for individuals with occupational HBV exposure. Internationally, lower thresholds (e.g., ≥10 IU/L) are often considered sufficient for protection. However, due to legal requirements in Germany, we adhered to the Standing Committee on Vaccination guidelines in this study. A more detailed discussion of these international differences is provided in the discussion section).

### Occupational group differences

To investigate whether infection risk self-assessment varied by occupation (e.g., teachers vs. educational specialists), we analyzed responses from the online anamnesis form. An “overall risk of infection” scale was developed, assigning values (0–3) to each response option (“never,” “rarely,” “occasionally,” and “often”). The total score, ranging from 0 to 24, represented the overall infection risk. To assess for significant differences between occupational groups, we utilized the Kruskal–Wallis test, as the data did not meet the assumption of normal distribution. For post-hoc comparisons, Mann–Whitney *U* tests with Bonferroni correction were applied, considering *p* < 0.017 as statistically significant. Effect size (𝑟) was calculated by dividing the *z*-value by the square root of the sample size (*r* = *Z*/√*N*). Effect sizes were classified as small (0.1), medium (0.3), or large (0.5).

Chi-square tests were conducted to assess the association between HAV and HBV immunostatus and occupational groups. For significant chi-square test results, post-hoc analyses were conducted using multiple *z*-tests for proportions with Bonferroni correction, considering *p* < 0.006 as statistically significant to identify differing groups. To identify personal and professional factors influencing the decision to vaccinate against HAV/HBV following a doctor’s recommendation, a binary logistic regression analysis was conducted, with vaccination status (yes/no) as the dependent variable. The analysis included age, gender, occupational group, general health status, and the “total risk of infection” scale. Adjusted odds ratios (aOR) with 95% confidence intervals (95% CI) were calculated as effect measures, with a significance level set at *α* = 0.05. Descriptive analysis was performed using Excel for Windows 2016, while statistical analysis was conducted with SPSS® 29.

### Data protection and ethics vote

The data presented in this publication was collected via the online anamnesis form, medical anamnesis, and on-site occupational health care examinations. All data was anonymized and does not allow identification of individuals. Data collection and processing complied with the requirements of the data protection officer of the University Medical Center Mainz. The study analyzes routine data collected by the Institute for Teachers’ Health, which are presented in anonymized form. After consultation with the responsible ethics committee of Rhineland-Palatinate and a review of the study design and medical history questionnaires, the committee confirmed that no further approval was needed. Thus, the study was approved by the ethics committee of Rhineland-Palatinate. Participants were informed about the use of data collected during mandatory occupational health screenings, including its analysis for scientific purposes. Accordingly, informed consent was obtained. We confirm that all research was performed in accordance with relevant guidelines and regulations.

## Results

### Study cohort

Between April 2021 and November 2023 a total of 1,742 employees at special schools in Rhineland-Palatinate participated in occupational health screening (see Table 2).

**Table 2:** Gender, age, and professional group of the study cohort.

Variable	*n*	%
Gender		
Female	1,455	83.5
Male	287	16.5
Age (Mean, Min–Max, SD)	43.7 (18–71, 12.0)	
Professional group		
Teacher	580	33.3
Educational specialists	1,083	62.2
Trainee teacher	38	2.2
Others^*^	41	2.4

* e.g., Early intervention specialists, physiotherapists, educators, trainees, interns, substitute teachers.

### General state of health

Participants self-rated their general state of health, with 70.8% (*n* = 891) reporting their general health as very good or good, and 29.2% (*n* = 368) rating it as fair, poor, or very poor.

### Self-assessment of occupational infection risk and exposure to biological agents

**Figure 1:**
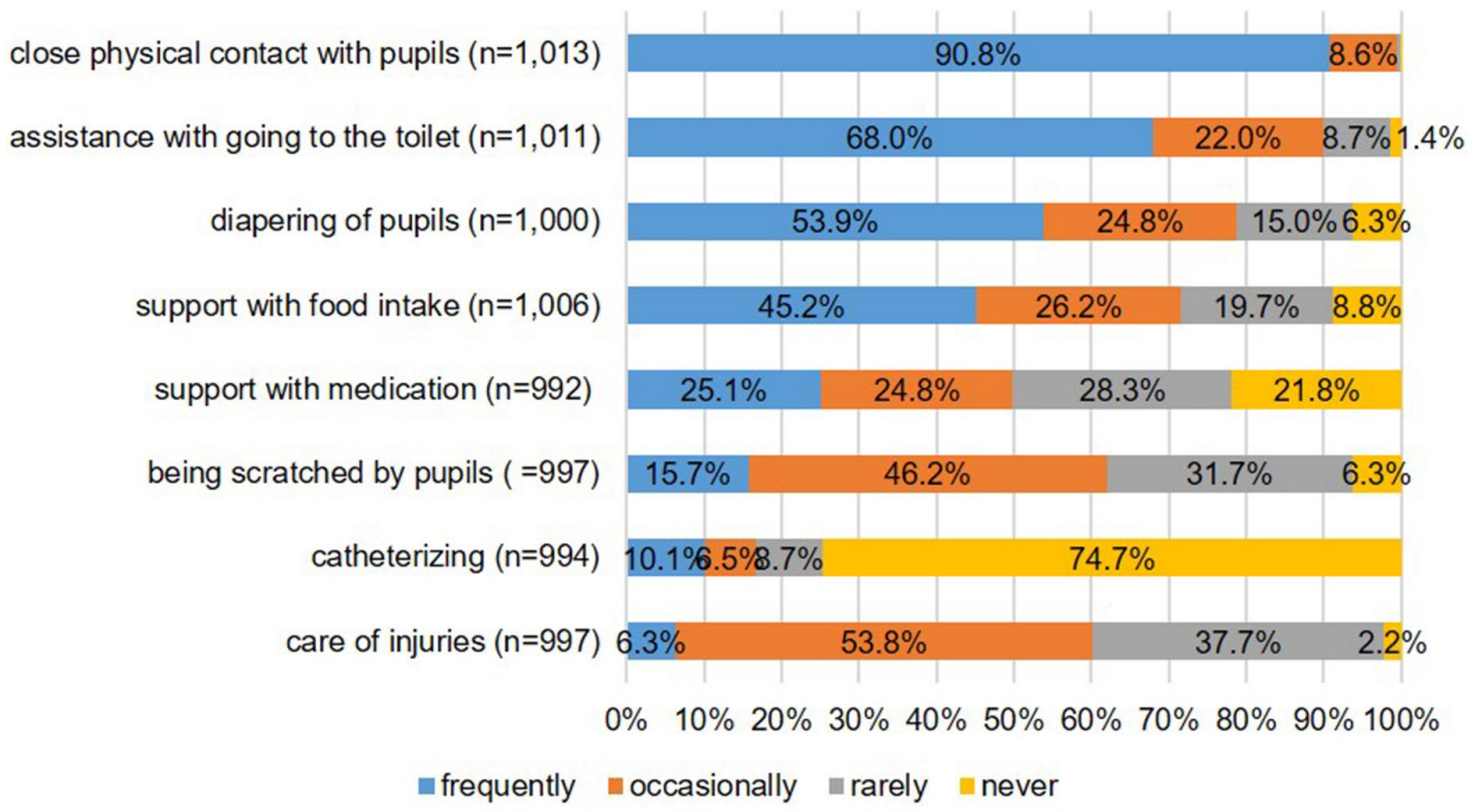
Data on self-assessment of occupational exposure to biological agents.

A total of 1,299 participants completed the online anamnesis form (74.6%), and 1,016 (78.2%) of these participants answered “yes” to the question about increased occupational infection risk. Following a more in-depth survey on potential infection risks, nearly all respondents stated they had frequent or occasional close physical contact with pupils. A large portion of those surveyed also reported regularly assisting pupils with toileting, washing, or changing diapers. Figure 1 provides more specific information on these and additional infection risks.

The majority (94.1%, *n* = 956) of those who reported an increased infection risk stated that they had access to suitable protective equipment, such as gloves and hand sanitizer, for care activities. However, 6.5% (*n* = 66) reported experiencing a needlestick injury or an injury in which the transmission of pathogens was possible in the course of their work.

### Self-assessment of occupational infection risk—differences between occupational groups

**Figure 2:**
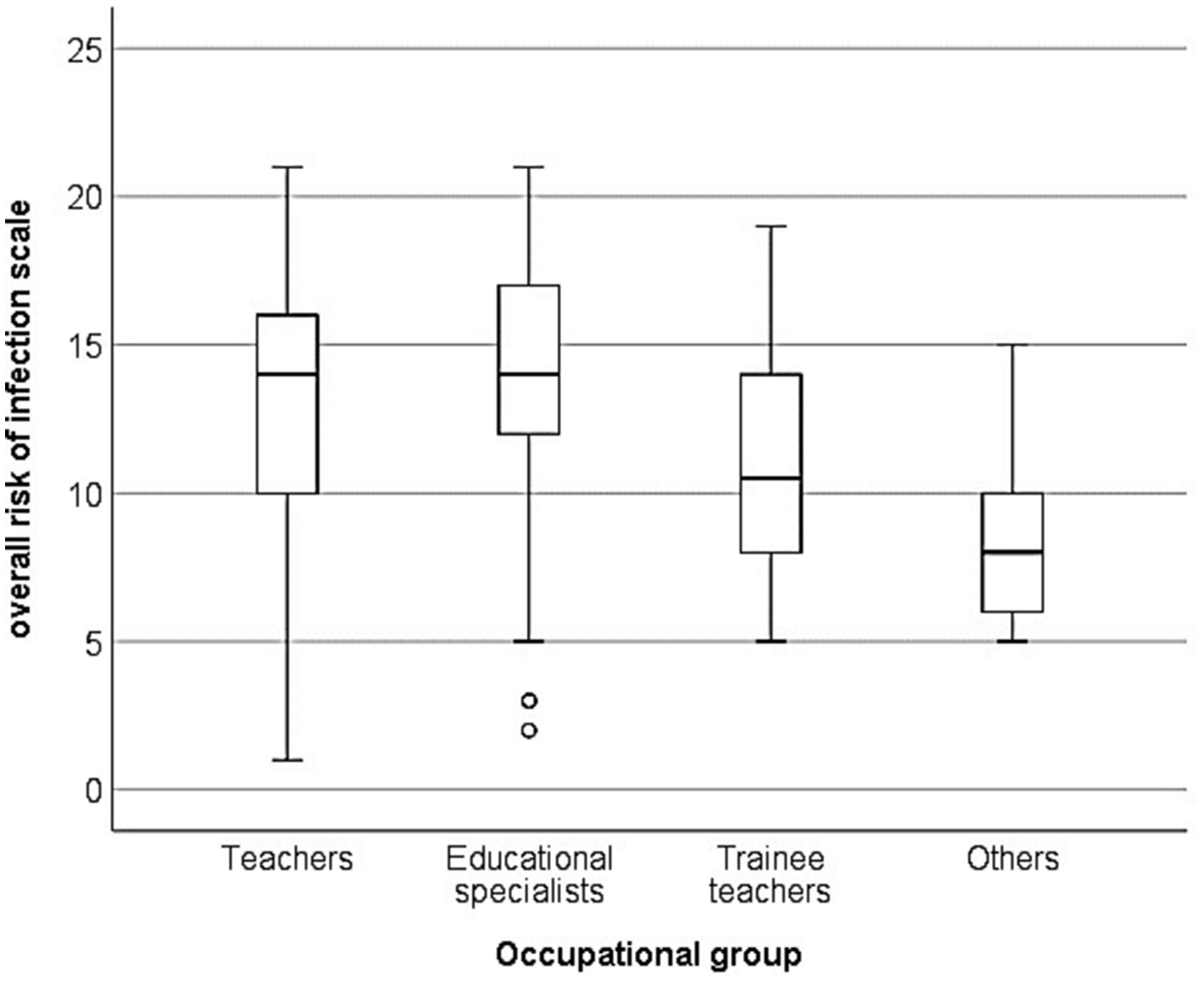
Self-assessment of occupational infection risk by occupational group.

Figure 2 shows the distribution of the overall risk of infection scale (self-assessment) by occupational group. The Kruskal–Wallis test revealed significant differences in the self-assessment of the occupational infection risk between the occupational groups (*χ*^2^(2) = 36.496, *p* < 0.001). *Post hoc* analyses showed that teachers reported significantly lower risk than educational specialists (*z* = −3.998, *p* < 0.001, *r* = 0.1) and that the “other” group reported significantly lower risks than both teachers (*z* = 3.142, *p* = 0.010, *r* = 0.1) and educational specialists (*z* = 3.987, *p* < 0.001, *r* = 0.1). The effect size (*r* = 0.1) indicates a small effect.

### Immunity against HAV and HBV

According to medical assessment, a little more than half of all participants (*n* = 940; 54.0%) had immunity to HAV, while 58.7% had immunity to HBV (*n* = 1,022). Among those who provided information on their immune status in the online anamnesis form a large proportion of respondents believed they were immune. Medical assessments based on vaccination records or serology confirmed immunity was lower (see Table 3).

**Table 3:** HAV and HBV immune status according to self-assessment and medical assessment (vaccination records or serological testing).

Immune status	Immune status according to self-assessment	Immune status according to medical assessment
HAV (*n* = 183)	88.0%	71.0%
HBV (*n* = 213)	92.5%	72.3%

Despite self-reporting immunity in the online anamnesis form, 19.9% (*n* = 32) of those reporting HAV immunity and 29.5% (*n* = 58) of those reporting HBV immunity received vaccination recommendations following medical assessment.

### Vaccination recommendations and vaccinations

Table 4 details the frequency of vaccination recommendations made during the occupational health care visits. Of all participants, a total of 1,010 (58.0%) received a vaccination recommendation, while the others did not. The table further specifies which vaccinations (HAV, HBV, or both) were recommended and illustrates the corresponding acceptance rates among employees.

**Table 4:** Frequency of vaccination on the date of the occupational health care visit.

Vaccination recommendation	Vaccination				Overall
	HAV (%)	HBV (%)	HAV/HBV (%)	No vaccination (%)	
HAV	61 (48.4%)	0	0	65 (51.6%)	126
HBV	0	186 (62.4%)	0	112 (37.6%)	298
HAV/HBV	3 (0.5%)	3 (0.5%)	328 (56.0%)	252 (43.0%)	586
No vaccination recommended	0	0	0	732	732
Overall	64	189	328	1,161	1,742

Of the 1,010 individuals who received the vaccination recommendation, 581 (57.5%) accepted and were vaccinated on the day of the occupational health care visit, while 429 (42.5%) declined. As of December 2024, follow-up data on these 429 individuals indicate that 63 (3.6%) were subsequently vaccinated, while the vaccination status of the remaining 366 was incomplete.

### HAV and HBV immunity—differences between occupational groups

**Figure 3:**
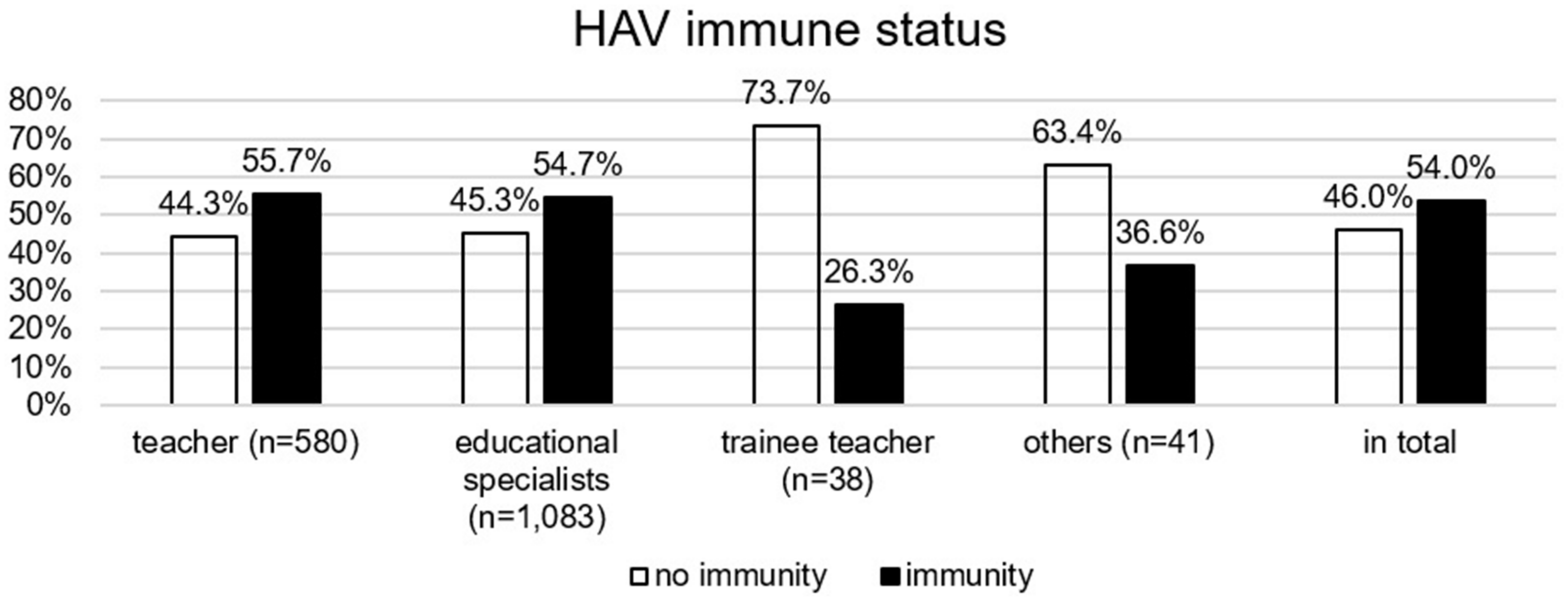
HAV immune status (based on vaccination records or serological testing) by occupational group.

**Figure 4:**
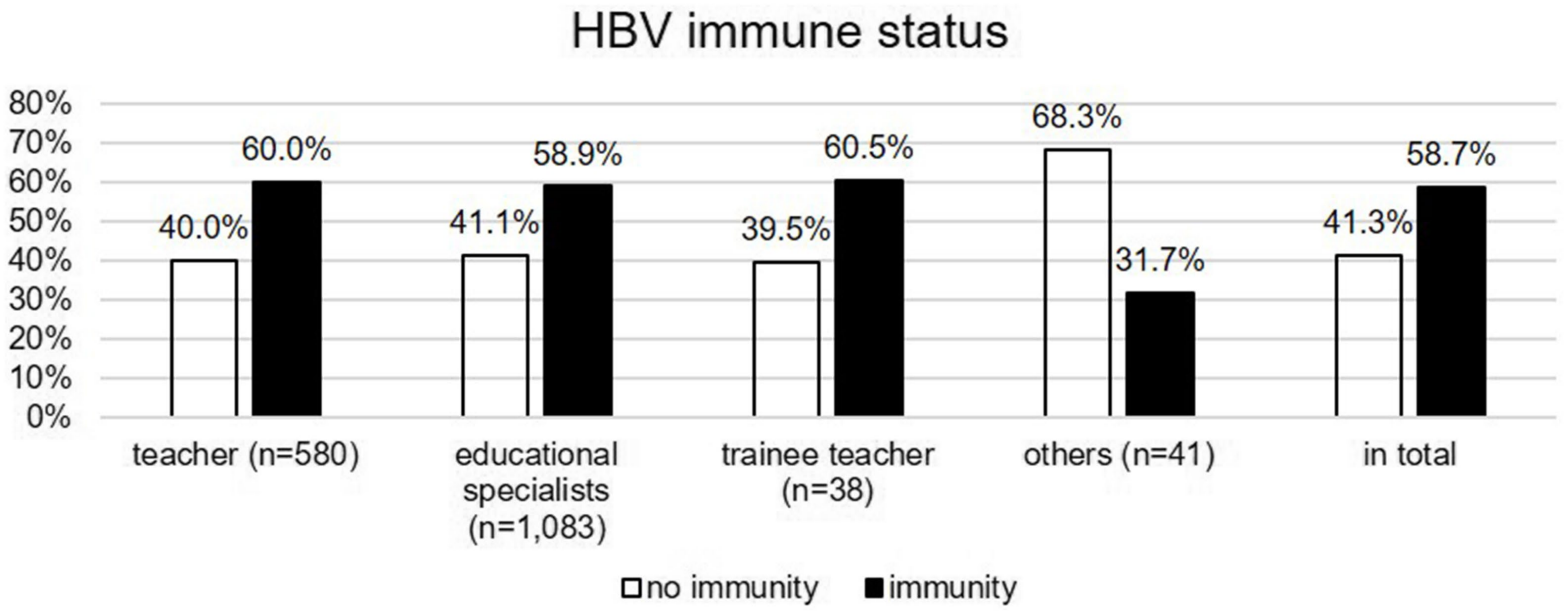
HBV immune status (based on vaccination records or serological testing) by occupational group.

Figures 3, 4 illustrate HAV and HBV immunity status by occupational group, based on vaccination records or serological testing. Just over half of teachers and educational specialists were immune to HAV, while significantly fewer trainee teachers and individuals in the “other” category had immunity (*χ*^2^(3) = 17.585, *p* = 0.021). *Post hoc* analyses revealed that trainee teachers were significantly less likely to be immunized against HAV than the other occupational groups. Teachers, educational specialists, and trainees were significantly more frequently immunized against HBV than members the “other” group (*χ*^2^(3) = 12.795, *p* = 0.005), a finding further confirmed by *post hoc* tests.

### Logistic regression

A logistic regression analysis was conducted to assess the influence of personal and professional factors on adherence to vaccination recommendations for HAV and/or HBV. Of the 1,010 individuals who received a vaccination recommendation, 528 were included in the regression model, which was statistically significant (*χ*^2^(7) = 19.240, *p* < 0.007, *R*^2^ = 0.050).

Among the five variables examined, only age had a significant effect (*p* < 0.001), while gender, general health status, occupational group, and infection risk were not significant predictors (Table 5). Age was negatively associated with adherence to vaccination recommendations, with an odds ratio of 0.968 (95% CI [0.952, 0.985]), meaning that for each additional year of age, the likelihood of accepting the vaccination recommendation decreased by 3.2%.

**Table 5:** Results of logistic regression.

Variable	*B*	SE	Wald	*p*	Odds ratio	95% CI for odds ratio	
						Lower bound	Upper bound
Gender	0.33	0.28	1.41	0.235	1.391	0.807	2.400
Age	−0.03	0.01	14.01	<0.001	0.968	0.952	0.985
General health status	0.25	0.22	1.31	0.253	1.279	0.839	1.951
Teachers			2.71	0.438			
Educational specialists	−0.11	0.21	0.26	0.612	0.897	0.590	1.364
Trainee teacher	−1.03	0.63	2.65	0.103	0.359	0.105	1.231
Others	0.01	0.92	0.000	0.996	1.005	0.167	6.038
Infection risk	0.03	0.03	1.06	0.304	1.028	0.976	1.083
Constant	1.79	0.55	10.47	0.001			

CI, Confidence Intervalls.

## Discussion

### Risk perception

This study demonstrates that employees at special schools perceive themselves to be at elevated occupational risk of HAV and HBV infection. This perception is particularly evident among those engaged in activities involving close physical contact, such as assisting pupils with toileting, nappy changing, catheterisation, administering medication, treating injuries or assaults, and managing incidents related to aggression. Teachers assessed their risk significantly lower than educational specialists. Those in the ‘other’ group reported the lowest risk. These variations may stem from differences in job responsibilities, the frequency of direct student contact, and differing risk perception thresholds. While the effect sizes in *post hoc* comparisons were small, the occupational group differences are still relevant from a practical point of view. The findings suggest that tailored prevention measures addressing the specific risks and needs of different professional groups could yield optimal outcomes.

Among the participants, 6.5% reported workplace injuries involving a risk of infection, such as needlestick injuries. This confirms findings from previous surveys on infection risks (1, 2). Encouragingly, most employees reported having access to the appropriate protective equipment and hand sanitisers for their caregiving duties.

### HAV and HBV-immunity and vaccination gaps

Even though precise risk indicators are lacking, based on the literature it can be assumed that employees at special schools have an increased risk of HBV infection (3, 4). According to the literature, disabled children also have an increased prevalence of hepatitis C (3, 5). Based on our risk assessment, we assume that the HAV and HBV infection risk is partly comparable to that of healthcare workers. For this professional group, HBV vaccination has been recommended by the Standing Committee on Vaccination since the early 1980s, including booster doses when the anti-HBs titer declines (10). Given these factors, occupational health care and appropriate HAV and HBV immunization protection are essential for ensuring workplace safety in special schools.

Compared with available data, HAV immunity in our cohort is broadly consistent with the German population average (11), while HBV immunity is higher than in the general population but considerably lower than among healthcare workers (12, 13). These protection gaps are relevant, since daily care activities at special schools involve close contact with body fluids, partly resembling risks in healthcare. None of the professional groups examined reached the World Health Organization’s (WHO) target of over 95% HBV vaccination coverage (14). In Germany, the HBV vaccination has been recommended by the Standing Committee on Vaccination for infants since 1995. Therefore, a large proportion of the people examined here are not yet covered by this regulation, but have either not received a HBV vaccination or have received one at a later age. It is to be expected that the HBV vaccination recommendation for infants will improve the vaccination status in the future, also in the occupational group considered here. Nevertheless the results of this study highlight the current need for targeted vaccination campaigns in this area. International recommendations for HBV protection vary. While the WHO considers antibody titers of ≥10 IU/L to be sufficient, and some guidelines assume that complete primary immunization provides lifelong protection (15, 16), Standing Committee on Vaccination requires a titer of ≥100 IU/L for individuals with occupational exposure. The Standing Committee on Vaccination also recommends monitoring antibody titers and administering booster doses if an adequate immune response cannot be verified (9). Our study applied this stricter definition, which is legally binding for occupational health practices in Germany. This likely explains the lower reported immunity compared with international figures, respectively, makes international comparison difficult.

In the study cohort, there was a discrepancy between self-reported HAV and HBV immunity and actual immunity as confirmed by vaccination records or serology tests. This underlines the importance of medical assessments, timely occupational health consultations, and readily available vaccination services. Even medical professionals sometimes misjudge their immune status, including misinterpretations of previous vaccinations and past infections (17).

More than half of the participants received a vaccination recommendation, most often for the combined HAV/HBV vaccine. However, acceptance was only moderate (~57%), indicating that many employees declined vaccination despite medical advice. Follow-up visits to respective schools resulted in only a modest (~4%) increase in vaccine uptake. This suggests that extending the decision-making time does not substantially improve acceptance. Reasons for the low vaccination rates may include a lack of information about the importance of vaccination or concerns about side effects. Additionally, the data collection period coincided largely with the SARS-CoV-2 pandemic, during which concerns about interactions with the SARS-CoV-2 vaccine or infection were common. However, the reasons for refusing the vaccine were not systematically assessed, which represents a clear limitation of this study and indicates the need for further research.

### Factors influencing vaccination acceptance

Logistic regression analysis indicated that age had a significant influence on non-compliance with vaccination recommendations, whereas gender, health status, professional role, and perceived occupational infection risk had no significant impact. The likelihood of accepting the vaccination recommendation decreased with age. Results from other studies have shown that vaccination rates for HAV and HBV vary widely based on country, age, and profession (18). Higher occupational risk (e.g., among healthcare and social workers) or a travel history are positively associated with greater vaccine acceptance, while chronically ill or older individuals are more likely to decline vaccination (12). Therefore, it is possible that older people assess the risk of infection at work as lower than younger people do. Additionally, older individuals may be more sceptical about vaccinations, whereas younger people are more likely to accept them as ‘standard prevention’. Furthermore, younger people are more accustomed to vaccination programmes, whereas older people tend to have more catching up to do.

In summary, our study shows that occupational health care at special schools is both reasonable and necessary, given the existing vaccination gaps and inaccurate self-assessment of hepatitis A and B immunity. At the same time, further research into the reasons for vaccine refusal is required to enable targeted improvements to counseling and education. Reasons for vaccine refusal to develop more tailored educational interventions.

### Limitations

The data presented in this study was collected from employees at special schools for disabled pupils in Rhineland-Palatinate who underwent mandatory occupational health care assessments based on risk evaluations. Therefore, vaccination and immunity findings may not be generalizable to all teachers. Furthermore the data on the vaccination status cannot be directly compared with the general population not exposed to HBV in the workplace, as stricter criteria for vaccination status apply in Germany for people exposed to HBV in the workplace (basic immunization, anti-HBs titer once higher than 100 IU/L, otherwise booster vaccination) than for the general population. The study used a pragmatic, self-developed scale to assess occupational infection risk. The items were derived from prior surveys conducted by the Institute for Teachers’ Health in 2012 and 2016. The scale was not formally validated, but its descriptive results are presented for transparency (Figure 1). Additionally, subgroup sample sizes were relatively small, which should be considered when interpreting the results. The survey period coincided with the SARS-CoV-2 pandemic, which may have introduced biases in vaccine acceptance due to concerns about interactions or perceived contraindications. This study did not assess the reasons for vaccine refusal, which represents a major limitation and highlights the need for future research.

## Data Availability

The raw data supporting the conclusions of this article will be made available by the authors, without undue reservation.
